# Public health reasoning: much more than deduction

**DOI:** 10.1186/0778-7367-71-25

**Published:** 2013-09-18

**Authors:** Louise Cummings

**Affiliations:** 1 Nottingham Trent University, Nottingham, UK

## Abstract

The deductive paradigm has produced notable successes in epidemiology and public health. But while deductive logic has made a substantial contribution to the public health field, it must be recognized that there are also limits to that contribution. This report examines one such limit: the need for non-deductive models in public health reasoning. The findings of a study of public health reasoning in 879 members of the public are reported. Four non-deductive strategies were chosen for their capacity to bridge gaps in one’s knowledge. It emerged that subjects were adept at using these strategies in the absence of knowledge to arrive at judgements about public health problems. The implications of this finding for public health communication are discussed.

## Background

Deductive reasoning is a powerful logical resource. Through valid inferences from true premises, deductive reasoning carries a guarantee that a derived conclusion is not only certain to be true but should be viewed as such by any rational person. But deductive reasoning is limited in one important respect. Notwithstanding its dominance in logic, deductive reasoning plays a somewhat minor role in the various deliberations which feature in our daily lives. From financial and moral problem-solving to decisions about our health and wellbeing, deductive reasoning is largely displaced during these deliberations by forms of reasoning which rarely receive serious treatment in logic textbooks. These non-deductive reasoning strategies, which include arguing in circles and from a lack of knowledge, prioritise effective problem-solving and other forms of decision-making over the deductive validity and soundness of arguments. De Beaugrande and Dressler (1981: 93–94) state that ‘[h]umans are evidently capable of intricate reasoning processes that traditional logics cannot explain: jumping to conclusions, pursuing subjective analogies, and even reasoning in the absence of knowledge....The important standard here is not that such a procedure is logically unsound, but rather that the procedures work well enough in everyday affairs’ [1]. In this short report, I describe the findings of a study of public health reasoning in 879 members of the public. This study explored the use of four non-deductive reasoning strategies in the context of public health deliberations. The findings reveal that people are adept at using non-deductive reasoning strategies during complex problem-solving in matters of public health. We begin with a brief examination of the strategies in question, and consider their role in public health reasoning.

## The limits of deduction

Deduction involves linear, unidirectional reasoning typically from one or two premises to a single conclusion. The premises and conclusions of deductive arguments are variously characterized as true, certain or known propositions. Deductive reasoning is particularly suited to domains in which theses or statements have the status of certainties or are at least well-established propositions. But many (or most) areas of enquiry lack the type of propositional certainties which are the essence of deduction. Instead, people tend to reason from premises which have the status of presumptions, using inferences which are non-linear and bidirectional in nature (e.g. circular reasoning) [2]. Public health, it is contended, is one domain in which non-deductive reasoning from presumptive premises predominates. On account of a lack of knowledge and other epistemic limitations, people employ reasoning strategies about public health problems which differ markedly from deduction. Four such strategies include arguing from ignorance and from authority, and the use of circular and analogical reasoning. Each of these reasoning strategies is well adapted to the public health context on account of its ability to bridge gaps in one’s knowledge. This important heuristic function of these strategies has been discussed in several publications to date [3-9]. For the purposes of the current discussion, this function is demonstrated in the case of one of these strategies, the argument from ignorance. In a classic argument from ignorance, one argues from an absence of knowledge or evidence that *P* is true to the conclusion that *P* is false (also, from an absence of knowledge or evidence that *P* is false to the conclusion that *P* is true). The former version of this argument has the following form:

There is *no evidence* that scrapie is transmissible to humans.

Therefore, scrapie is *not* transmissible to humans.

The standard logical view of the argument from ignorance is that it is an informal fallacy – a lack of knowledge, it is contended, is a weak basis indeed for a conclusion about the truth or falsity of a proposition. However, in more recent logical analyses this argument is considered to be not only non-fallacious but strongly rationally warranted under certain epistemic conditions. These conditions include the *epistemic closure* of a knowledge base in a particular domain and the *exhaustive search* of that base. These conditions were fulfilled in the case of the above argument from ignorance which came to prominence during the UK’s BSE epidemic. This argument represented the conclusion of a study by Brown and colleagues in 1987 [10]. Certain attributes of this study conferred strong rational warrant on the conclusion of the argument. Specifically, a 15-year epidemiological investigation of scrapie (a brain disease in sheep) and Creutzfeldt-Jakob disease (CJD) in France enabled investigators to claim closure of the knowledge base on these diseases. Additionally, a review of the world literature by Brown and his colleagues allowed them to claim that this knowledge base had also been exhaustively searched. Under these epistemic conditions, a lack of evidence was a strong rational basis for the conclusion that scrapie is not transmissible to humans. This conclusion had considerable significance for scientists who later used it extensively in BSE risk assessments. (Most of these assessments were arrived at through an analogy with scrapie.) The heuristic value of this argument from ignorance lay in its use of an absence of knowledge or evidence to arrive at a conclusion about the non-transmissibility of scrapie to humans. The other non-deductive strategies examined in the current study have similar heuristic functions. The reader is referred to Gigerenzer and Brighton (2009) for discussion of the nature and function of heuristics [11].

## Study of public health reasoning

A total of 879 members of the public participated in a questionnaire study of public health reasoning. Subjects, who were both male and female and aged between 18 and 65 years, were from diverse educational, socioeconomic and ethnic backgrounds (see Table 1). All subjects completed an anonymous postal questionnaire in which a number of (actual and non-actual) public health scenarios were presented (see Table 2). Several epistemic conditions, which were expected to confer differing levels of rational warrant on the four non-deductive reasoning strategies, were systematically manipulated across these scenarios. Four questions followed each passage. Two of these questions could be answered on the basis of information explicitly presented in the passage. These questions were intended to create the impression among subjects that they were participating in a reading comprehension task. A third question examined if a particular inference had been drawn. A fourth question was open-ended and encouraged subjects to elaborate on the grounds for their response to the inference question. All passages were scrutinised by two public health consultants in advance of the study, and were considered to have high plausibility. Two linguists also examined the passages and assessed them to be comprehensible to the average reader.

**Table 1:** Subject characteristics

	**Subject characteristics (total = 879 subjects)**
**Age**	Average: 43.8 years
	Range: 18–65 years
**Gender**	Male: 292 subjects
	Female: 587 subjects
**Education**	University level: 589 subjects
	Secondary school level: 290 subjects
**Ethnicity**	White British: 789 subjects
	White Irish: 30 subjects
	Asian or British Asian Indian: 15 subjects
	Asian or British Asian Pakistani: 4 subjects
	Black or Black British Caribbean: 3 subjects
	Black or Black British African: 3 subjects
	Mixed: White and Black Caribbean: 1 subject
	Mixed: White and Black African: 1 subject
	Mixed: White and Asian: 1 subject
	Other: 32 subjects

**Table 2:** Public health scenarios

	**Description of public health scenario**
	***Arguments from authority:***
**1**	Pronouncements on BSE by the Spongiform Encephalopathy Advisory Committee
**2**	Use of chemicals in food production
**3**	Aspirin use and Reye’s syndrome in children
**4**	Cancer risks posed by a nuclear power facility
**5**	Safety of the measles, mumps and rubella (MMR) vaccine
**6**	Electromagnetic emissions from mobile phone masts
**7**	Pronouncements on BSE by the Southwood Working Party
**8**	Air-borne chemical emissions from a recycling facility
	***Arguments from ignorance:***
**9**	Risk assessment of the transmissibility of scrapie to humans
**10**	Assessment of findings from clinical trials of a new asthma drug
**11**	Risk assessment of the transmissibility of BSE to humans
**12**	Health risks associated with chemicals in effluent from a pharmaceutical plant
**13**	Assessment of the safety of genetically modified foods
**14**	Assessment of the safety of a food additive in dairy products
**15**	Safety of swine flu immunization
**16**	Location of the source of an outbreak of severe food poisoning
	***Analogical argument:***
**17**	The use of hepatitis B by the CDC in the US as a model for HIV/AIDS health advice
**18**	Investigation by epidemiologists of illness related to chemicals in drinking water
**19**	Use of scrapie by British scientists to assess the risk of BSE to human health
**20**	A study by epidemiologists of the health effects of a new arthritis drug
	***Circular argument:***
**21**	Investigation of fever in patients following vaccination for pneumonia
**22**	Investigation of a disease outbreak in the Congo by scientists from WHO
**23**	Study of a purported link between electromagnetic radiation and birth defects
**24**	Discovery of a novel disease by medical anthropologists working in Peru

Full results of the study are reported elsewhere [12]. Quantitative and qualitative data analyses indicated that subjects are adept at recognising the epistemic conditions under which all four non-deductive reasoning strategies are rationally warranted. Judgements about the problems presented in the scenarios revealed that conditions such as epistemic closure and exhaustive search hold logical sway for subjects. Subjects were aware of the conditions which precluded the closure of a knowledge base, tainted the opinion of an authority and justified the use of circles and analogies in reasoning. Moreover, they exercised these logical judgements in ways which were proportionate to the epistemic conditions which confronted investigators. Qualitative comments revealed several intuitive pragmatic criteria which were influential in subjects’ reasoning such as the cost in human health of delays in implementing public health measures. These criteria were taken to warrant a form of reasoning which was less oriented to achieving certain and true conclusions than the effective and timely implementation of public health actions. Judgement-making was not affected by the age, gender or education of subjects and was only minimally influenced by the presence of actual versus non-actual public health scenarios.

## Discussion

This investigation has revealed a rational competence on the part of humans that has been occluded from view by the traditional dominance of deduction in logic. This competence embodies a number of non-deductive reasoning strategies which serve humans well in contexts that are characterised by epistemic uncertainty. Public health is just such a context. When knowledge and evidence are lacking, the rational cognitive agent can ill-afford to suspend judgement until such times as evidence is forthcoming. Rather, this agent must draw upon cognitive strategies which fall short of a deductive logical standard in reasoning but which are nevertheless effective procedures (or heuristics) for arriving at solutions to problems. In serving to bridge gaps in our knowledge of public health problems, the four forms of reasoning examined in this study can be seen to function as cognitive heuristics. Through use of these strategies, subjects were able to arrive at *some* decision about the public health problems that confronted them rather than make *no* decision about these problems *at all*. These strategies can be added to a growing list of heuristics which is currently under investigation by cognitive scientists. Although these heuristics vary in their structure and function, they are all evolutionary adaptations of our rational resources to the problems of uncertainty and a lack of knowledge in the cognitive sphere.

## Conclusion

This study has shown that subjects are adept at using non-deductive reasoning strategies in their deliberations about public health problems. One implication of this study is that public health workers should seek to exploit this rational resource wherever possible. The most obvious way in which this can be achieved is during communication with the public. All too often during public health communication, members of the public are presented with recommendations in the absence of the wider epistemic context which is the rational basis of those recommendations. There is a mistaken assumption that because the public lacks expert knowledge of public health issues, they also lack a rational capacity to judge those issues. If this study demonstrates anything it is that members of the public are reasonably skilled in the manipulation of non-deductive reasoning strategies. The challenge now is for public health workers to integrate these strategies into increasingly effective forms of communication with the public.

## Ethical approval

Full ethical approval for this study was obtained from Nottingham Trent University, UK.

## Competing interests

The author declared that she has no competing interest.
